# Improvement of pain experience and changes in heart rate variability through music-imaginative pain treatment

**DOI:** 10.3389/fpain.2022.943360

**Published:** 2022-08-10

**Authors:** Susanne Metzner, Marc N. Jarczok, Irina Böckelmann, Sina Glomb, Manuela Delhey, Harald Gündel, Jörg Frommer

**Affiliations:** ^1^Faculty of Philosophy and Social Sciences, Augsburg University, Augsburg, Germany; ^2^Faculty of Medicine, Augsburg University, Augsburg, Germany; ^3^Department of Psychosomatic Medicine and Psychotherapy, University Hospital Magdeburg, Otto-von-Guericke-University Magdeburg, Magdeburg, Germany; ^4^Department of Psychosomatic Medicine and Psychotherapy, Ulm University Medical Centre, Ulm, Germany; ^5^Department of Occupational Medicine, Medical Faculty, Otto-von-Guericke-Universität Magdeburg, Magdeburg, Germany

**Keywords:** psychosomatics, somatoform pain disorder, music-imaginative pain treatment, heart rate variability, pain perception

## Abstract

Music-imaginative Pain Treatment (MIPT) is a form of music therapy addressing pain experience and affective attitudes toward pain. It includes two self-composed music pieces: one dedicated to the pain experience (pain music, PM) and the other to healing imagination (healing music, HM). Our non-experimental study addresses patients with chronic somatoform pain disorders participating in MIPT. The goal is to gain insight into the direct effect mechanisms of MIPT by combining outcome measures on both the objective physiological and subjective perception levels. The research questions are directed toward changes in pain experience and heart rate variability and their correlations. Thirty-seven hospitalized patients with chronic or somatoform pain disorders receiving MIPT participated in this study. Demographic data and psychometric measures (Symptom Check List SCL90, Childhood Trauma Questionnaire CTQ) were collected to characterize the sample. Subjective pain experience was measured by McGill Pain Questionnaire (SF-MPQ), and Heart Rate Variability by 24 h-ECG. Data analysis shows a reduction of reported pain from M_T1_ = 19.1 (SD = 7.3) to M_T2_ = 10.6 (SD = 8.0) in all dimensions of the SF-MPQ. HRV analyses shows a reduced absolute power during PM and HM, while a relative shift in the autonomic system toward higher vagal activity appears during HM. Significant correlations between HRV and MPQ could not be calculated. Findings are interpreted as a physiological correlate to the psychological processes of the patients. Future studies with more participants, a control-group design, and the integration of medium- and long-term effects are recommended.

## Introduction

### Music-imaginative pain treatment for chronic pain

In the context of pain treatment common music interventions utilize the simple listening to recorded music with the aim to enhance relaxation or distraction in painful situations. In contrast to this, professional music therapy focusses on psychic or interactive processes set in motion by playing music or listening to it. This corresponds to a more active involvement through emotional engagement and cognitive reflection within a therapeutic relationship. This approach is particularly relevant for chronic pain disorders.

In Germany, music therapy has a long tradition as part of the multi-professional treatment of hospitalized patients in departments for psychosomatic medicine (1). According to the national S3-guideline on “non-specific, functional and somatoform physical complaints” (2), music therapy is mentioned as a viable accompanying therapy approach, particularly for severe courses of disease. In this context, Music-imaginative Pain Treatment (MIPT) is an intervention that proves to be increasingly successful. It was initially developed as “entrainment” within a single session (3, 4) and later established as a manualized treatment by Metzner (5).

MIPT makes use of live music. In a room equipped with various musical instruments, the patient develops two pieces of music with the assistance of a trained music therapist. The first composition corresponds to the patient's experience of pain (“pain music”), while the other explores the ideas, or imaginations, of relief (“healing music”). Thereafter the therapist performs the two pieces of music, one after the other, as the patient is listening (application phase). MIPT involves a trusting, supportive therapeutic relationship and thorough verbal processing of what has appeared during the therapy sessions.

By presenting the two compositions one after another in the application phase a shift of pain experience in terms of intensity can be observed but predominantly in terms of the pain's qualitative character. Hauck et al. (6) found different patterns of neuronal activations in healthy subjects depending on which of the two compositions were presented. This correlated with the likewise different pain ratings during listening to PM and HM. Therefore, MIPT flexibilises the pain experience and furthermore the affective attitudes toward the pain. It increases the feeling of self-efficacy and promotes communication skills (7). The modification of the pain experience can be appropriately explained as a 2-folded approach. On one side, a transmodal process links affective-sensory pain with auditive music experience; on the other side, an imaginative activity leads to an assignment of musical symbols to pain (5).

Empirical research of MIPT has focused either on the activation of neuronal processes by MEG resp. EEG-measures (6, 8) or on therapeutic processes by qualitative studies (9, 10). Up to now, outcome studies (3, 11, 12) show promising results, but they are not transferable to patients with somatoform pain disorders, and existing systematic reviews do not include studies on this clientele (13–16).

### Chronic pain, music and heart rate variability

As music can influence heart rate variability (HRV) (17–19), not solely psychosocial but also (neuro-) physiological effects of MIPT can be assumed. HRV is considered an indicator of autonomic regulation/counter-regulation in patients with chronic pain disorders. Already in 1992, Gebhart and Rendich (20) assumed that the vagal afferents are an integral component of endogenous pain control systems. Koenig et al. (21) conducted a systematic review and meta-analysis on seven studies investigating group differences in vagally mediated HRV-Parameters in patients with headache disorders. The HRV-Parameters RMSSD and HF were reduced, but the authors emphasized the need for further research, as meta-regression analyses on covariates revealed significant differences by clinical etiology, age, gender, and length of HRV recording.

The spectral indices of cardiovascular autonomic control as measured by the spectral analysis of heart period and the mean systolic arterial pressure in women with Fibromyalgia syndrome seem to present good relative reliability. The FMS patients exhibited reduced activation of the sympathetic nervous system (in the LF power, heart rate, and mean arterial pressure) (22). However, a previous study (23) does not indicate dysregulation of spontaneous baroreflex sensitivity.

### Goals and objectives of the present study

Our study addresses patients with chronic somatoform pain disorders participating in MIPT. The primary goal is to gain insight into the direct effect mechanisms of MIPT by combining outcome measures on both the objective physiological and subjective perception levels. Our non-experimental study is considered a first step toward collecting quantitative data under naturalistic conditions. Findings of interrelationships between physiological changes during MIPT and short-time positive effects in pain ratings would form the basis for an extensive RCT.

### Hypotheses and research questions

Based on clinical observations, we expected to observe a reduction of sensory and affective pain experience after MIPT. Further, we were concerned with whether this is reflected on a physiological level by an increase in parasympathetic activity. Specifically, the research questions of our study were as follows: 1. Does the experience of pain improve between the start (T1) and the end of MIPT (T2)? 2. Is it possible to measure different response reactions in the autonomic nervous system when the patient listens to his/her two music pieces? 3. Are pain ratings at T2 correlated with changes in HRV-Parameters during MIPT? Based on clinical experience and evidence-based data from HRV changes in healthy subjects, we hypothesized:

H1–Subjective pain experienced at the beginning of MIPT (T1) is significantly improved after listening to the two music pieces (T2).
H2–Parasympathetic activity significantly increases while listening to the second music piece (“healing music”).
H3–Increased parasympathetic activity while listening to the second music piece (“healing music”) correlates negatively to the reduction of pain experienced after MIPT (T2).

## Methods and materials

### Study design and participants

This study recruited participants consecutively between 09/2016 and 10/2020 from hospitalized pain patients at the Department of Psychosomatic Medicine and Psychotherapy, University Hospital, Otto-von-Guericke-University Magdeburg, and the Department of Psychosomatic Medicine and Psychotherapy, University Medical Centre Ulm, both in Germany. After the specialist assessment, all patients who met the inclusion criteria were referred to MIPT within the first week of hospitalization. Inclusion criteria were the following primary ICD-10 diagnoses: Chronic pain disorder (F45.40 and F45.41) or somatoform pain disorders (F45.0, F45.1, F45.2, F45.3, F45.8, F45.9). Since this study uses a naturalistic study design, patients who took special heart medication (beta-blockers, ACE inhibitors, antiarrhythmics) or psychotropic drugs and analgesics, including opiates, were not excluded. Medication was systematically documented and taken into account in the data analysis. Exclusion criteria were a diagnosis of acute cardiac arrhythmias and previous participation in MIPT.

The study was approved by the ethics committees of the University Hospital and Medical Faculty of Otto von-Guericke-University Magdeburg (File reference: 179/16) and the University Medical Center Ulm (File reference: 201/18). It was carried out following the recommendation of the ICH-GCP guidelines, Declaration of Helsinki. All participants gave written informed consent before participation.

### MIPT-intervention

The participants received MIPT in the initial phase of the inpatient stay as the only music-therapeutic intervention. The intervention follows the manualized four treatment steps, which are usually divided into three sessions of 50 min each (5): 1. structured pain interview; 2. “composition phase”: creating a music piece addressing the pain experience (“pain music” PM) and another music piece addressing imaginations of relief (“healing music” HM); 3. “application phase”: live performance of the self-composed music pieces by the music therapist to the patient combined with 4. subsequent verbal reflection.

**Figure 1:**
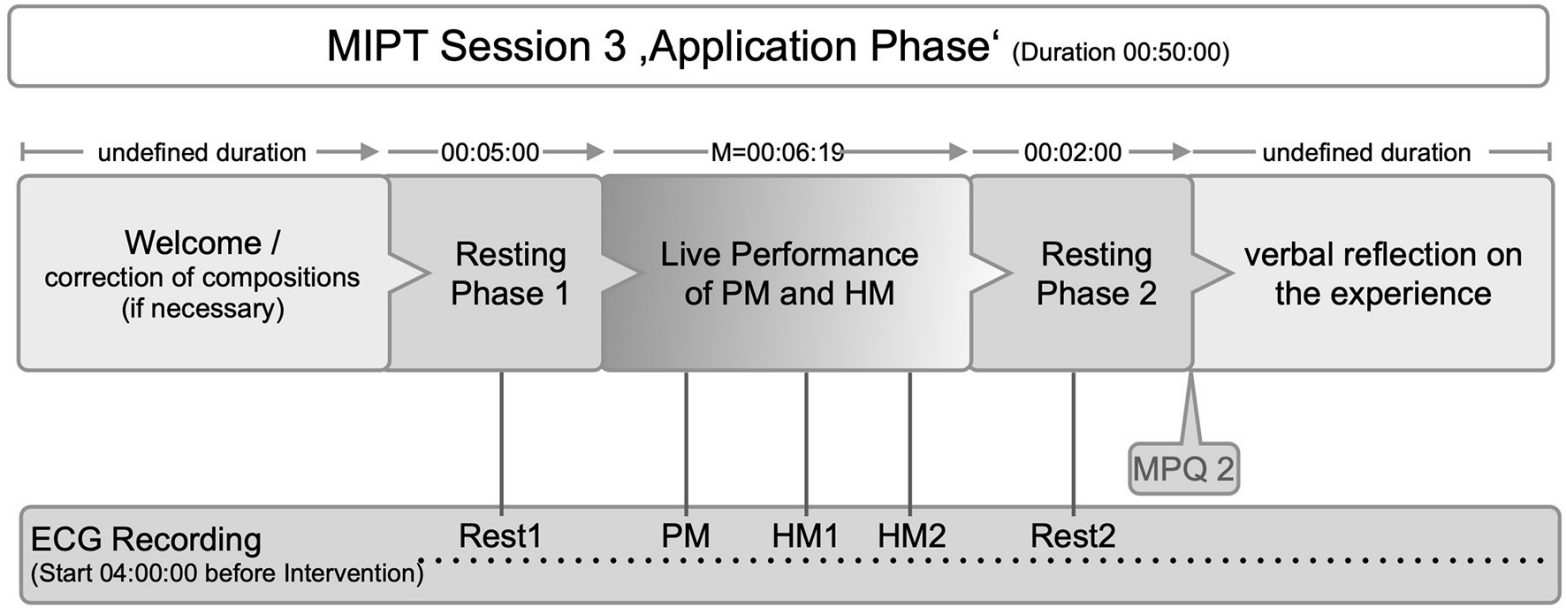
Course of the intervention (ECG, Electrocardiogramm; MPQ 2, McGill Pain Questionaire at T2; PM, pain music; HM, healing music; Rest1/PM/HM1/HM2/Rest2, time points for HRV analyses).

Figure 1 shows the course of intervention as follows: Patients were equipped with an ECG-Holter monitor (see below) at a minimum of 4 h before the 3rd MIPT-session (“application phase”) to accommodate the device. The intervention started with a greeting, a short reflection on the current state of health and on PM's and HM's veracity. Then, patients were seated in a comfortable position. Hand signals were agreed upon before the arrangement of the music. They serve as indicators for the beginning, end, tempo, and dynamics because there is no conversation during the music intervention itself. After a resting phase, participants listened to the music pieces performed by the music therapist.

### Demographic data, psychometric measures, music measures

Basic demographic items and potential confounding variables (shift work, chronic medication with influence on the heart rhythm, diabetes mellitus, untreated thyroid diseases and treated thyroid diseases with thyroid blood parameters outside the normal range, cardiac diseases, use of nocturnal oxygen or nightly continuous positive airway pressure) were collected with the MIGA questionnaire (24).

To capture the burden of symptoms in the intervention group immediately after admission to the hospital (T0), we used the following questionnaires: Symptom Check List (SCL90) (25) with 90 items and the German version of the Childhood Trauma Questionnaire (CTQ) (26) with 25 items. The German short version of the McGill Pain Questionnaire (SF-MPQ) (27) measures the subjective pain experience before (T1) and after (T2) MIPT. It records the sensory component (sum of 11 items) and the affective pain qualities (sum of four items).

**Figure 2:**
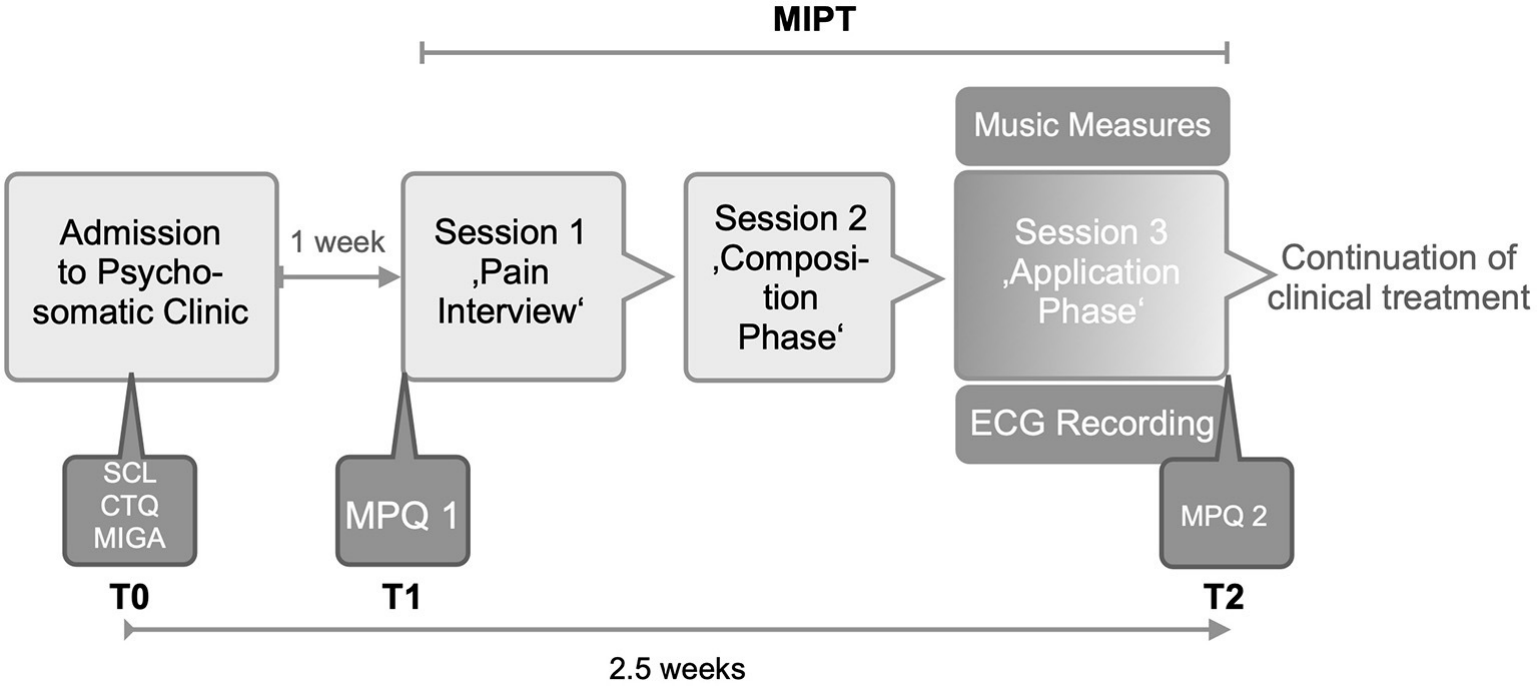
Study design (MIPT, Music-imaginative Pain Treatment; SCL, Symptom Check List; CTQ, Childhood Trauma Questionnaire; MIGA, MIGA Questionnaire; MPQ, McGill Pain Questionnaire; ECG, Electrocardiogramm).

Live performances of PM and HM during the “application phase” were audiotaped. The duration of the music was calculated to the second. Figure 2 shows the timepoints for the psychometric and musical data collection.

### Physiological measures

The basis for the HRV analysis were 24-h ECG recordings (model: MT-101 or Medilog^®^ AR12 PLUS Schiller AG, Baar, Switzerland) from voluntary patients who had participated in pain therapy. For this purpose, a 2-channel ECG recording at a sampling rate of 1,000 Hz is stored on the SD card located in the Holter ECG. The raw data RR time series (NN-Interval resp. Normal beat-to-Normal beat) were transferred to the Medilog^®^ DARWIN2 Enterprise analysis software package for the total recording time (artifact correction). The data were checked automatically and visually by a healthcare professional for clinical abnormalities and converted into text files comprising consecutive NN intervals. The subjects completed an activity protocol to note the activities in the following 24 h while wearing the ECG.

The HRV parameters were calculated with the Kubios HRV 3.4.3 software (University of Eastern Finland, Kuopio, Finland). The measuring NN intervals and the HRV analysis correspond to the quality criteria recommendations according to the national and international guidelines (28, 29).

Taking the ECG measurement into account, some experimental modifications had to be introduced, i.e., 5-min rest periods before and after the music. The composition representing the pain experience (PM) is played for at least 2 min, and the composition representing the idea of alleviation (HM) is played immediately afterward for at least 5 min. If the respective music pieces fall below the specified time periods, participants are to be regarded as dropouts due to a lack of evaluability on the HRV markers.

Artifact correction was done with an artifact identification threshold of 0.3 s and a smoothness prior method for detrending NN intervals (Lambda = 500, f_c_ = 0.035 Hz). Mean RR and HRV parameters from time and frequency domains were calculated. Calculated time domain parameters were the standard deviation of NN intervals (SDNN) and the root mean square of successive RR interval difference (RMSSD) in milliseconds. The following frequency domain parameters were calculated using the autoregressive methods (AR): Total power (TP), the low frequency (LF) from 0.04 to 0.15 Hz, and high frequency (HF) from 0.15 to 0.40 Hz. Additionally, the relative power of the HF band (%) was calculated for each experimental phase. Because the shortest experimental phase has a duration of 2 min, the Total Power consisted primarily of HF and LF.

The experimental phases are a resting period before program start (Rest 1), “pain music” period (PM), “healing music” period (HM 1) with first 2 min and last 2 min (HM 2), and resting period at the end (Rest 2) (see Figure 1).

### Statistical analysis

All data were checked for normality using the Shapiro Wilk‘s test. The hypotheses were tested using *t*-tests for paired (within-subject) and independent (between-group) samples. Similarly, a non-parametric test was applied for paired (Wilcoxon sign rank) and independent (Wilcoxon rank-sum aka Mann-Whitney U test) samples to confirm parametric results. Statistical significance was set to *p* < 0.05 two-sided. All statistical analyses were calculated using Stata v15.1 SE (Stata Corp. College Station, Texas).

## Results

### Sample

Initially, 51 participants were recruited. This number was reduced by 14 dropouts (1 weak health condition, one refusal of study participation, three early treatment termination, nine insufficient ECG data). No causal connection to music therapy was reported. The final sample comprised 37 participants with chronic or somatoform pain disorders who engaged in MIPT for the duration of the study. A balanced distribution of participants between the two clinics could not be achieved due to the different resources. Therefore, 64.4 % of the sample comes from Magdeburg.

### Demographic data, psychometric measures, music measures

Table 1 provides an overview of the demographic data, psychometric and musical measures, that characterize our sample. The mean age of all participants was 50 years (SD = 10.9), 33% were diagnosed with Chronic pain disorder (ICD-10: F45.40 and F45.41). The symptom burden of our sample, measured with SCL 90, showed high values in Global Severity: 1.4 (SD = 0.7). The CTQ total mean (*n* = 36) was M = 55.6 (SD = 11.5). The prevalence in the subscales was highest for the emotional abuse M = 11.6 (6.6), emotional neglect M = 16.3 (6.1) and physical neglect M = 10.7 (2.7).

**Table 1:** Demographic Data, psychometric measures, and musical data: SCL90, Symptom Check List; GSI, Global Severity Index; CTQ, Childhood Trauma Questionnaire.

**Number of participants**	***N*** = **37** **(Magdeburg n1** = **26;** **Ulm n2** = **11)**			
**Demographic data**
**Mean age (years)**	49.6 (SD 10.7)			
**Female**	28	76%		
**Occupation**
Full- or part-time employed	13	35%		
Registered unemployed	11	29%		
Unable to work for 6–24 weeks	25	66%		
Early retirement/disability pension	12	33%		
Unknown	1	2%		
**Current smoking**	21	56%		
**Psychometric measures**	**mean (SD)**	**median**	**min**	**max**
**SCL90 GSI (*****N*** **=** **36)**	1.4 (0.7)	1.4	0.35	3.3
**GSI T-value>60 (** ***N*** **, %)**	*N* = 28	80%		
**CTQ total score (*****N*** **=** **36)**	55.6 (11.5)	39	95	53 d
**Music measures**				
**Mean music duration (*****N*** **=** **36)**	06:19 (SD 01:44)		

A total number of each 36 PM and 36 HM have been composed. The mean duration of combined PM and HM during the application phase was 06:19 min (SD 01:44). The musical characteristics varied according to the subjective pain experience and imagination of relief. Two examples of PM and HM are provided as Supplementary material.

### Outcome variables

#### Pain ratings

All patients that were included in the study completed the full questionnaires. Cronbach's alpha on the sensory scale was 0.67 (T0) and 0.82 (T1). Cronbach's alpha on the affective scale was 0.52 (T0) and 0.71 (T1). Table 2 shows that there is a reduction of reported pain from M_T1_ = 19.1 (SD = 7.3) to M_T2_ = 10.6 (SD = 8.0) and in all dimensions of the MPQ-SF. These data confirm our first hypothesis stating a significant reduction of sensory and affective pain experience shortly after the completion of MIPT.

**Table 2:** Pain ratings at two timepoints measured by McGill Pain Questionnaire (German Short Form).

		**Pain ratings at T1**					**Pain ratings at T2**						
**MPQ SF**	***N***	**Mean**	**SD**	**Min**	**Max**	**p50**	**Mean**	**SD**	**Min**	**Max**	**p50**	**T-test *p***	**Wilcoxon sign rank *p***
												**2-sided**	**2-sided**
Sensory (Ia)	32	12.6	5.9	4	26	13	7.6	5.9	0	28	7	<0.001	<0.001
Affective (Ib)	32	6.8	2.5	2	11	6.5	3.2	2.8	0	11	2.5	<0.001	<0.001
Total (Ia+Ib)	32	19.3	7.2	9	33	18.5	10.8	8.4	0	39	10	<0.001	<0.001
VAS	30	6.8	2	1	10	7	4	2.8	0	10	4	<0.001	<0.001
Pain perception now	31	2.4	1.4	0	5	3	1.7	1	0	3	2	<0.001	<0.001

#### Physiological measures

Since the HRV data may have high interindividual variability and different reactivity, the baseline data were determined beforehand as a starting point to perform the individual normalization. The HRV during resting measurements is subject to only a few influences. As mentioned in the sample section, there were dropouts due to insufficient ECG data. Experimental periods with an artifact rate of 5% or higher were excluded from the analysis (nine periods in three patients). Six patients had an ECG that could not be analyzed due to high age and diagnose-related ectopic beats. Table 3 shows the cardiologic baseline measures of the sample.

**Table 3:** Cardiologic baseline measures.

**HRV parameter baseline**	**Mean (SD)**	**Median**	**Min**	**Max**
Mean RR (ms)	761 (120)	561	1,050	752
SDNN (ms)	21.8 (13.7)	6.06	68.6	18.5
RMSSD (ms)	16.3 (12.1)	3.08	53.5	14.7
PNN50 (%)	3.14 (7.6)	0	34.8	0
LF (ms^2^)	397 (609)	18.1	2,821	113
HF (ms^2^)	150 (299)	2.83	1,547	69.4
Relative LF (%)	59.8 (20.1)	10.9	86.9	63.4
Relative HF (%)	26.2 (22.2)	4.15	87.4	18.9
Total power (ms^2^)	615 (919)	29.5	4,743	243

The short analysis phases were selected to investigate the mean differences of the Mean RR and HRV parameters during the test phases “pain music” (PM) and “healing music” (HM1). Table 4 documents physiological data during PM and HM1 2 min after each piece of music have started.

**Table 4:** Comparison of Mean RR and HRV parameters in the phase during Pain Music (PM) and Healing Music (HM1).

		**Pain music (PM)**					**Healing music (HM1)**						
**Variable**	***N***	**Mean**	**SD**	**Min**	**Max**	**p50**	**Mean**	**SD**	**Min**	**Max**	**p50**	***T*-test *p***	**Wilcoxon sign rank *p***
												**2-sided**	**2-sided**
Mean RR (ms)	36	772	109	599	1,088	763	772	122	606	1,100	760	>0.10	>0.10
SDNN (ms)	36	22.3	14.8	6.2	72.5	17.5	18.8	11.2	5.2	58.8	15.8	0.051	0.011
RMSSD (ms)	36	16.0	10.6	2.8	46.0	13.9	14.9	10.3	3.0	49.5	12.9	>0.10	>0.10
LF (ms^2^)	36	471	808	13	4,161	173	282	462	5	2,629	118	>0.10	0.011
HF (ms^2^)	36	106.0	146.0	1.9	724.0	57.6	108.0	154.0	2.6	724.0	62.3	>0.10	>0.10
Relative LF (%)	36	63.7	17.1	24.5	91.8	67.2	61.1	16.4	22.2	84.5	63.4	>0.10	>0.10
Relative HF (%)	36	22.9	15.8	3.4	62.3	19.2	27.9	16.3	5.5	63.5	26.4	0.021	0.029
Total power (ms^2^)	36	644	948	33	4,789	281	432	608	23	3,294	209	>0.10	0.029

The HRV parameter Total Power (TP), which reflects the overall variability, is reduced (*p* = 0.0288) in the phase of the HM (644.0 ms; SD = 948.0 ms vs. 432.0 ms; SD = 608.0 ms^2^). The parameter Total Power reflects the overall level of the autonomic-regulation status, which means that the regulation status is lower during HM. Even when using a correction factor, the comparability of the determined absolute frequency powers seems to be limited due to the strong spread. The standard deviations (SD) are larger than the mean values of these parameters.

**Figure 3:**
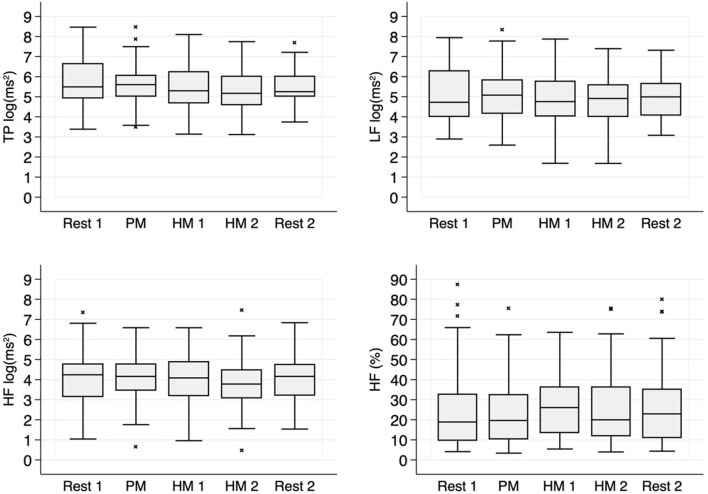
HRV-Parameter from frequency domain: Rest: Resting period before (1; *N* = 37) and after (2; *N* = 37) music, PM: Pain Music (*N* = 37), HM: Healing Music first 2 min (1; *N* = 36) and last 2 min (2; *N* = 36). The sample size differs due to a shorter duration of HM in one case.

The relative Power (%) in the HF band (HF-Power %) is completely different. The HF band reflects parasympathetic activity. It's relative power increases during HM1 [22.9% (SD = 15.8%) vs. 27.9% (SD = 16.3%); *p* = 0.0288]. Figure 3 shows boxplots of a 2-min study phase for four selected HRV parameters.

The modulation of the parasympathetic tone helps to maintain the dynamic vegetative regulation. The absolute power in this respiratory band does not differ in these two phases. So, while the absolute power is reduced in TP and HF, there appears to be a relative shift in the autonomic system toward higher vagal activity.

#### Correlation between painratings and HRV-data

Although negative correlations between the change of HRV parameters from PM-HM1 with the actual pain experience were observed in this sample, the parametric and non-parametric correlation coefficients were small and not significant.

## Discussion

While in our study a significant reduction in pain experience before (T1) and after (T2) MIPT was found, the data on heart rate variability during the “application phase” of MIPT were much more complex than expected. Significant correlations between the two variables could not be calculated. These findings require more detailed analysis and interpretation in the following.

### Study relevance: Challenges in the treatment of chronic somatoform pain disorders

Chronic pain is a multidimensional phenomenon related to somatic illness, personal life history, psychological vulnerability, and interpersonal relationships. A particular therapeutic challenge is posed by the group of patients with chronic somatoform pain disorder included in our study. This clientele has a somatic understanding of illness predominantly. Demographic data of the study participants revealed a precarious professional situation, while the psychometric data showed a particularly increased symptom burden. Furthermore, our sample is characterized by childhood traumatization, notably emotional abuse and neglect. The psychologically harsh living conditions in childhood can lead to a lack of ability to differentiate between physical pain and affects (30), and can be accompanied by incoherent verbal and nonverbal communication (31). This gives reason to integrate music therapy as a partly nonverbal element of Germany's multimodal inpatient treatment of chronic pain.

### Improvement of pain experience

Our measures show a significant reduction in the pain ratings in a period of < 2 weeks, and our first hypothesis, H1, can therefore be confirmed. However, the improvement cannot be attributed to our intervention alone, as MIPT is an element of a multimodal inpatient treatment concept and because of lack of a control condition. Other studies on MIPT with comparable clients are not yet available, not even when applying another music therapy intervention. Therefore, we cannot relate our results to available data sets.

Considering that long standstills often characterize the treatment of chronic somatoform pain disorders, our results are nevertheless remarkable. Even minor improvements can be interpreted as a positive sign that an intervention has started working. The contribution of MIPT we regard as a music-induced change in pain perception. Compared to music therapy approaches that use music for relaxation, mindfulness, or distraction, patients in MIPT are encouraged to engage with their pain experience actively and directly. By creating a musical product and communicating in this way, subjective pain experience becomes an object of perception, resulting in perceptual structures and habituated attitudes changing. Not only moments of altered pain perception in the initial phase of the inpatient stay but experiences of self-efficacy are decisive for the treatment progress, as they raise the patients' hope and increase motivation.

### Physiological data

The role of vagal afferents in the modulation of pain is established. A systematic review by Koenig et al. (32) includes 20 studies on HRV in healthy adults with experimentally induced pain. It shows an increase in sympathetic-baroreflex activity indexed by an increase in low frequency (LF) spectrum and a decrease in vagal-parasympathetic activity indexed by a decrease in high frequency (HF) spectrum. Healthy individuals with self-reported pain symptoms may have lower parasympathetic activity, indexed by pNN05, RMSSD, and HF (33).

Our HRV data found a more complex situation than has been found in music intervention studies with healthy people. In our view, this might be due to fundamental differences between healthy and severely ill persons. The clinical picture of chronic pain patients is often associated with pronounced vegetative accompanying symptoms in addition to the pain symptoms. The pain perception includes the hypothalamic-pituitary-adrenal axis (HPA axis) with involvement of the autonomic nervous system (ANS) (23). This demonstrates a reduced ability to be activated and adapted to stressful situations in chronic pain patients. People with reduced sympathetic activity show a deficit of pain-inhibiting mechanisms with an increased pain perception (34). On this basis, it is remarkable that the measured HRV changes refer to very low durations of the music in our study. Despite a manifest baseline situation, the immediate effect of listening to the self-composed music can be observed well between the measuring time points during the application phase of MIPT.

We explain our findings psychologically because there is no physiological reference data so far. In the beginning, the HRV parameter Total Power (TP) remains approximately the same during the “pain music” compared to the resting phase, probably caused by tension when anticipating what is about to come. In the phase of “healing music,” TP, mainly comprising of HF and LF, decreased. As exciting music decreases the HRV (17), HM can no longer be understood as relaxation music but as one that can be thrilling for the patient with chronic pain. Interestingly, when HM started and TP tendentially decreased, HF percentage increased. This indicates a shift toward lower mixed sympathetic and parasympathetic activity. We interpret our findings as a physiological correlate of the psychological work of the patients, which, generally speaking, consists in resolving the conflict between the desire for healing and the fear of change. Given the complexity of the physiological data and the lack of significance, our hypotheses H2 and H3 cannot be confirmed.

### Limitations and outlook

The small sample size and the lack of a control group limit the validity of our results. The high dispersion of data does not allow a statement about general tendencies of physiological reactions to the intervention. We attribute this to the characteristics of our naturalistic study and the range of individual propensities within our sample. Different cardiologic and psychopathological starting points could have been the reason for different reactions to the music therapy treatment. This might explain why a significant correlation between HRV and MPQ could not be calculated.

Our present study is unique so far. Although our results are only indicative, they have raised research questions for future studies. They also provide insight into immediate physiological reactions to self-composed music pieces as well as into complex physiological and psychological interactions during MIPT. Therefore, our study has increased knowledge of individualized chronic pain treatment with an activating and partly confronting model of music therapy. In a follow-up study, we recommend including a control group and expanding to measurements of intermediate and longer-term impacts of MIPT.

## Data availability statement

The original contributions presented in the study are included in the article/Supplementary material, further inquiries can be directed to the corresponding author.

## Ethics statement

The studies involving human participants were reviewed and approved by University Hospital and Medical Faculty of Otto-von-Guericke-University Magdeburg (File reference: 179/16) University Medical Center Ulm (File reference: 201/18). The patients/participants provided their written informed consent to participate in this study.

## Author contributions

SM, JF, IB, and SG: conception and design. SG and MD: study therapists. MJ: statistical analysis plan. SM, IB, MJ, JF, and HG: first draft. All authors reviewed and edited the manuscript.

## Conflict of interest

The authors declare that the research was conducted in the absence of any commercial or financial relationships that could be construed as a potential conflict of interest.

## Publisher's note

All claims expressed in this article are solely those of the authors and do not necessarily represent those of their affiliated organizations, or those of the publisher, the editors and the reviewers. Any product that may be evaluated in this article, or claim that may be made by its manufacturer, is not guaranteed or endorsed by the publisher.

## Supplementary material

Supplementary Audios 1 and 2 Pain Music (PM).

Supplementary Audios 3 and 4 Healing Music (HM).
